# A TEC Cooling Soft Robot Driven by Twisted String Actuators

**DOI:** 10.3390/biomimetics8020221

**Published:** 2023-05-25

**Authors:** Shun Zhao, Xuewei Lu, Kunyang Wang, Di Zhao, Xu Wang, Lei Ren, Luquan Ren

**Affiliations:** 1 Key Laboratory of Bionic Engineering, Ministry of Education, Jilin University, Changchun 130025, China; 2 Weihai Institute for Bionics, Jilin University, Weihai 264402, China; 3 Institute of Structured and Architected Materials, Liaoning Academy of Materials, Shenyang 110167, China

**Keywords:** twisted polymer actuator, thermoelectric cooler, self-sensing, soft-hardware integration for robot systems

## Abstract

Similar to biological muscles in nature, artificial muscles have unique advantages for driving bionic robots. However, there is still a large gap between the performance of existing artificial muscles and biological muscles. Twisted polymer actuators (TPAs) convert rotary motion from torsional to linear motion. TPAs are known for their high energy efficiency and large linear strain and stress outputs. A simple, lightweight, low-cost, self-sensing robot powered using a TPA and cooled using a thermoelectric cooler (TEC) was proposed in this study. Because TPA burns easily at high temperatures, traditional soft robots driven by TPAs have low movement frequencies. In this study, a temperature sensor and TEC were combined to develop a closed-loop temperature control system to ensure that the internal temperature of the robot was 5 °C to cool the TPAs quickly. The robot could move at a frequency of 1 Hz. Moreover, a self-sensing soft robot was proposed based on the TPA contraction length and resistance. When the motion frequency was 0.01 Hz, the TPA had good self-sensing ability and the root-mean-square error of the angle of the soft robot was less than 3.89% of the measurement amplitude. This study not only proposed a new cooling method for improving the motion frequency of soft robots but also verified the autokinetic performance of the TPAs.

## 1. Introduction

Bionic robots inspired by animals comprise one of the most important research areas regarding robots. Soft robots are robots made of soft materials, such as silicone, rubber, and fabric [1]. They are designed to be more flexible and adaptive than traditional robots, allowing them to interact more naturally with their environment. Soft robots have several advantages over traditional robots [2]. They are more compliant than traditional robots and can conform to their environment, making them better suited for tasks that require delicate manipulation or contact with humans [3]. As the key driving component of soft robots, artificial muscle is a new type of intelligent driver that has been developed in recent years. Under the action of external stimuli (light, electricity, heat, humidity, etc.), artificial muscle can produce reversible contractions and bending, rotation, and deformation motions, as well as other complex actions [4].

Although soft robots have great innate advantages in the field of bionics, most of the existing bionic robots can only achieve a single movement function due to the limitation of the imperfect development of soft actuators. Compared with traditional rigid drivers, artificial muscles have higher flexibility, universality, and power density; these properties provide high strength, low noise, and a high-degree-of-freedom drive force, enabling the development of flexible mechanical systems [5]. Furthermore, artificial muscles can be combined with flexible electronic technology to realize integrated drive-sensing, leading to robots with improved characteristics [6]. Traditional artificial muscles, such as shape memory alloys (SMAs) [7,8], carbon nanotube (CNT) artificial muscles [9,10], and dielectric elastomer actuators (DEAs) [11,12] have the disadvantages of high production cost, large lag effects, short service life, and limited force and deformation output, which limit their use and applicability [13].

In 2014, Haines et al. repurposed polymer fibres designed for fishing and sewing thread into a novel twisted polymer actuator (TPA) for artificial muscle [14]. These materials could be used for artificial muscles in various applications, such as soft robots, biomedical devices, and smart textiles. TPAs are low-cost, easy to manufacture through a continuous process, and can be manufactured with a variety of precursor materials. The materials included nylon 6,6 composite silk sewing thread and nylon 6 monofilament fishing line. TPAs are characterized by low hysteresis effects, a long cycle life, low raw material and production costs, high energy density (up to 5.3 kJ/kg), and large output deformation (up to 49% maximum length shrinkage).

However, TPAs have several disadvantages that limit their application. An electric field or heat must be applied to the polymer material to drive motion. Compared with other actuators, its mechanism is relatively simple, but its response speed is slow and its response frequency is low. Furthermore, TPAs have distinct response frequencies depending on the heating and cooling media [15,16,17,18]. In one study, the maximum tracking frequency obtained via Joule heating and fan cooling was 0.15 Hz [19]. TPAs were used as antagonistic muscles, and different control modes and fan cooling were adopted to achieve a 0.1 Hz motion-tracking effect [20,21].

Cold and hot fluid reservoirs [22], water cooling [15,16], and forced convection air [23] can improve the TPA bandwidth. However, water cooling and forced convection air require additional systems to provide air or water flow, increasing the complexity and weight of the system. Furthermore, the use of hot and cold fluid reservoirs requires complex valves and pumps, temperature regulation mechanisms, and increases the possibility of fluid leakage.

To determine the shape of the soft robot, we usually need to measure the bending angle of the body [1,24]. While traditional solid-state sensors can measure bending angles, they have specific software requirements. Closed-loop control with TPAs uses encoders [19,25] as reliable sensors to feed back the control parameters of the robot in real time. Based on this, we designed an MPU6050 to provide feedback and a thermoelectric cooler (TEC) to provide cooling to achieve fast closed-loop control of a soft robot. In addition to the common sensors, the TPA sensor used by many researchers is another feasible embedded sensing method for soft robots [26,27,28,29]. The TPA sensor can sense its deformation through changes in resistance, allowing for the use of very simple resistance measurement circuits to provide geometry information feedback. In this study, we also tested the performance of the TPA as a sensor to sense the angle change of the robot.

A soft robot composed of four TPAs was designed by simulating the composition of human muscles in nature. Similar to the human muscle, except that the bionic muscle will contract during the actuation process, there is also a parallel antagonistic muscle effect to prevent the robot from transitional bending. In this work, we proposed a soft robot with TEC as the cooling system and a TPA as the sensor, which can realize fast responses and self-feedback control. The TEC has a fast response speed and can achieve rapid cooling in a few seconds, and high precision temperature control is achieved through current control. Moreover, the TEC is easy to install and needs to be in direct contact with only the radiator or the object to be cooled. The TEC facilitates the development of cooling systems that can be integrated into soft robots.

Our contributions can be summarized as follows:

1. In this study, a TEC and TPA were combined to develop a soft robot to improve the cooling effect of the TPA and the movement frequency of the robot. By testing different TPA cooling modes, we found that the TEC was easy to integrate and had a good cooling effect on the soft robot.
2. This study proposed control of the TPA through Simulink for the first time, making it convenient for new researchers to learn the robot’s operation. The TPA data, namely, the resistance value of the sensor, was collected by Simulink through MATLAB, and the corresponding deformation was obtained through mathematical model calculations to realize self-sensing. Feedback signals from an MPU6050 were transmitted to the TPA through an Arduino to provide feedback.

The remainder of this article is structured as follows. In Section 2, we introduce the materials and fabrication methods of soft robots. The experimental results that verified that the system improved the frequency and the effect of the robot’s self-sensing feedback angle change through the TPA are presented in Section 3. The discussion is presented in Section 4. We conclude the paper in Section 5.

## 2. Materials and Methods of the Soft Robot

### 2.1. Materials

#### 2.1.1. Manufacture

In the driving mode of the TPA, an artificial muscle coil with a spring-like structure produces contractions or elongation deformations when its temperature changes. Different spiral coil structures produce distinct deformation forms based on temperature changes.

**Figure 1:**
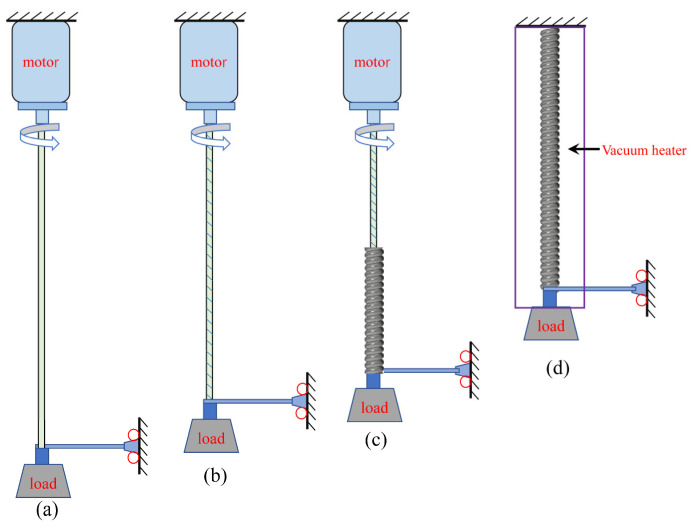
The production process of single-fibre winding to produce an artificial muscle: (**a**) initial state; (**b**) spiral after torsion of the TPA; (**c**) spiralling of the TPA; (**d**) heat treatment of the TPA.

In this work, we used silver-coated nylon 66 threads (235/36 dtex) as the raw material to produce the TPA. The production of a single TPA muscle (1 ply) is shown in Figure 1. A weight was hung on one end of the fibre, and the other end was connected to a rotating motor. The loads on the 1-ply and 2-ply muscles were 15 g and 20 g, respectively. The lengths of the fibres decreased as they were twisted (Figure 1a,b). When the twist increased to a certain critical value, the sewing thread became twisted and unstable and finally wound into a spiral structure (Figure 1c). Because twisting causes residual stress in the muscle, the fibre must be annealed, and annealing in a vacuum can prevent oxidation of the coating. The two 1-ply nylon wires could be processed into 2-ply muscles with additional winding, and then a heat treatment was performed. It should be noted that the direction of the motor rotation was the same in all winding processes, namely, clockwise.

Multiple coiled nylon wires provide more driving force than a 1-ply wire. However, finer strings can be heated faster via Joule heating, and thus, the process can be started faster, and they have higher resistance and are less likely to break due to excessive input power or overheating.

#### 2.1.2. Cooling

A TPA has an oriented length and significant anisotropic thermal expansion behaviour. Temperature changes can cause the volume of the spiral-wound TPA to increase, resulting in tensile or torsional motion. Artificial muscles made from polymers cannot be used directly for driving and require an external device as a heat source. In this study, Joule heating was used to heat the TPA, which generated contractions or rotational motion. After the TPA was shrunk via Joule heating, it needed to be cooled before the next heating contraction could occur, and a smaller duty cycle of the voltage pulse signal indicated a longer cooling time. When the TPA received the voltage pulse signal, it shrunk instantaneously, as the TPA bandwidth was 5 Hz [3]. Then, the TPA was held in air for tens of seconds before it was completely cooled for the next contraction cycle. Therefore, cooling was the most critical factor in increasing the frequency of the TPA.

Most researchers use water or air cooling to cool the TPA. In this study, the 2-ply muscle was cooled in water or cold air. The cold air approach involved an air flow generated by a mobile air conditioner. To obtain the maximum frequency under different cooling conditions, a 16 V signal was input under cold air conditions, and an 18 V signal was input in water after the test, where the maximum voltage value of the selected, as the TPA was not burned out. The difference between choosing different voltage values in water and air was because, even in plasma, there were still conductors in the water, and thus, a larger voltage value was required in water for the same length of TPA; when the power of the TPA is greater, it can bring about a higher contraction frequency. Moreover, 0.9 W/cm was applied in cold air, and 1.1 W/cm was applied in water to normalize the power of the connection and the length of the TPA. Under cold air conditions, the heating time was 100 ms, the cooling time was 200 ms, and the frequency was designed to be 3.3 Hz. Under water conditions, the heating time was 100 ms, the cooling time was 150 ms, and the frequency was designed to be 4 Hz. In addition, the carrying capacity of the 2-ply muscle was tested, and the carrying weight during the experiment was designed to be 400 g.

**Figure 2:**
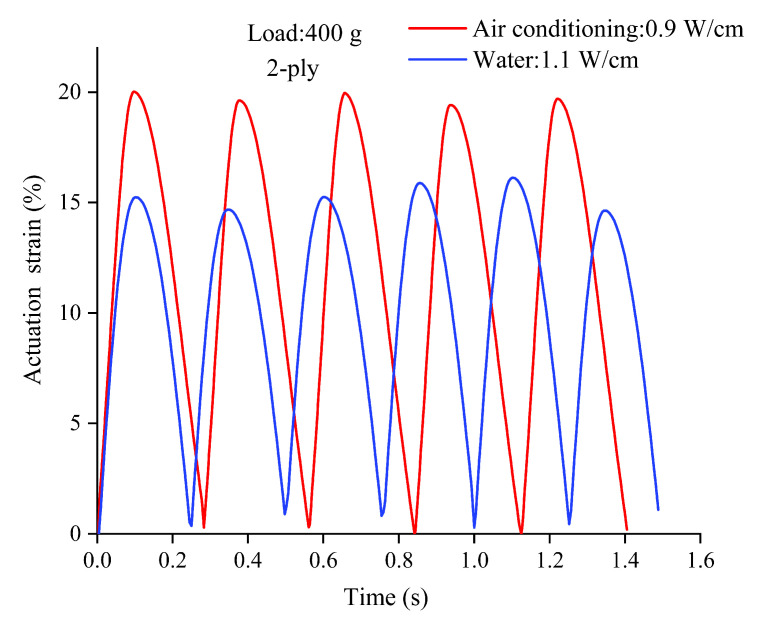
Contraction frequency under different cooling methods.

As shown in Figure 2, under the water condition at 5 °C, a contraction frequency of 4 Hz could be achieved in water refrigeration conditions, but the contraction and recovery of the TPA varied in different cycles, and the maximum shrinkage reached 15%. Under the condition of cold air refrigeration at 5 °C, a shrinkage frequency of 3.3 Hz could be achieved, and the shrinkage could reach 20%. In addition, the shrinkage and recovery of each cycle were essentially the same. When the cooling temperature was lower, the cooling time during the contraction movement could be reduced and the contraction frequency of the TPA could be increased.

Air cooling is easier to control than water cooling. However, water cooling and cold air refrigeration cooling both limit certain applications; for water cooling, the sealing, water circulation, and the water itself need to be considered, while air-conditioning refrigeration is not suitable for certain applications. Therefore, we adopted a semiconductor refrigeration sheet, which can reduce the internal air temperature of the small soft robot while improving the cooling effect.

#### 2.1.3. Sensor Characterization

**Figure 3:**
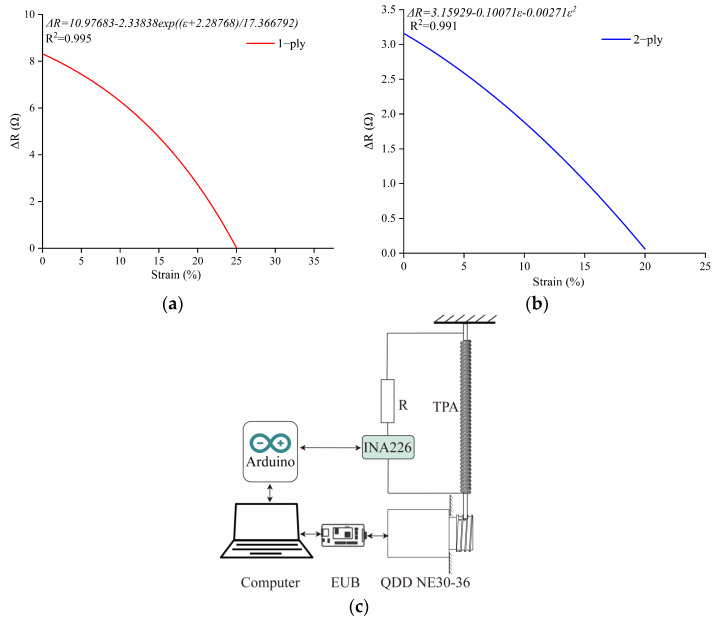
The resistance of the TPA varied with its length: (**a**) 1-ply; (**b**) 2-ply; (**c**) test diagram. Compared with the variation in the 1-ply resistivity during TPA shrinking, the 2-ply resistivity was more linear and had a smaller variation in the resistance value. The experimental results were linearly fitted through cubic polynomials, as shown in Formula (2). The 2-ply muscle also had a high coefficient of certainty and high repeatability.

A change in attitude can be perceived by the robot by measuring the resistance during TPA elongation, as shown in Figure 3c. In the characterization experiment, one end of the TPA was fixed to the base and the other end was connected to a brushless DC motor (QDD-NE30-36). The motor was controlled using a drive board (Ethernet to CAN Bridge), and the speed was 0.2 mm/s. Each experiment was repeated ten times. The resistance value of the silver-coated nylon 6.6 threads was 0.27 Ω/cm. The resistance of the TPA was tested using an INA226 and an Arduino.

In this study, we tested the resistance values of 1-ply and 2-ply muscles that varied with the contraction of the TPA. The test results are shown in Figure 3. As the test result of the 1-ply muscle was nonlinear, the Gauss—Newton method was adopted for nonlinear fitting and the fitting equation is shown in Formula (1). The experimental data had a high coefficient of certainty, with *R*^2^ = 0.995. The repeatability of the resistivity variation was verified.

(1) $$\Delta R_{1}=10.97683-2.33838^{\frac{\varepsilon +2.28768}{17.366792}}$$

where ∆*R*_1_ indicates the 1-ply resistivity change and *ε* indicates the TPA contraction ratio.

(2) $$\Delta R_{2}=3.15929-0.10071\varepsilon -0.00271\varepsilon^{2}$$

where ∆*R*_2_ indicates the 2-ply resistivity change.

This section describes the fabrication, cooling, and self-sensing characteristics of the TPA. The results show that the air cooling had a good effect on the contraction bandwidth of the TPA. Therefore, a TEC was proposed as the TPA refrigeration method to cool the internal air of the soft robot and improve its motion frequency. Furthermore, the results suggest that the 2-ply material had strong linearity and was easier to control as a self-sensing muscle. Thus, the 2-ply material was used as the muscle of the robot in the following design and tests.

### 2.2. Design and Control of the Soft Robot

To verify the performance of the proposed cooling method and the autokinetic properties of the TPA, we designed a system to measure the motion of the soft robot. The system consisted of two parts. First, the soft robot was driven by the TPA via Joule heating and TEC cooling, and the experimental conditions were designed to be as similar as possible to those in the actual application. Second, the system measured the actual angle, in addition to the perception of the angle change by the TPA, so that the angle induced by the TPA could be easily evaluated. The actual angle of the soft robot was obtained using an MPU6050.

#### 2.2.1. Manufacture

The complex motion of soft robots is generally realized using a series of bending action combinations with soft materials. Therefore, general modular soft robots must have the ability to bend. The body of the soft robot designed in this study was composed of soft materials and five channels. Four circular channels, arranged evenly around the software component at 90°, were used to place the 2-ply TPAs. At the same time, the TEC cooled the air inside these four channels. The square channel in the middle was placed in the centre of the software component and served as a place to hold the wires and thermometers. The soft robot was equipped with 3D-printed end covers at each end, which were used to hold the TPA and store the cold air inside the robot and the external circulation.

**Figure 4:**
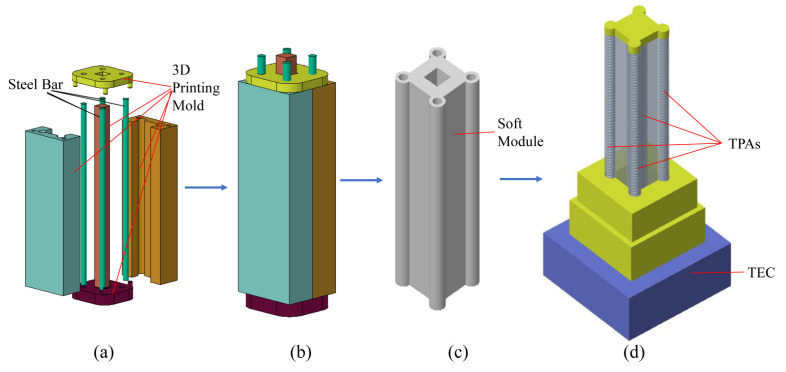
Manufacturing method of the soft robot embedded with four TPAs: (**a**) mould assembly; (**b**) assembly of the mould and filling the mould with silicone; (**c**) demoulding; (**d**) final soft robot.

The overall pouring process is shown in Figure 4. The mould for casting the silicone body consisted of 4 stainless steel rods and 3D-printed components. The steel bars were installed at the corresponding hole position in the mould and served as the core to make 5 channels in the body. After the mould was assembled, liquid silicone was injected into the mould, and the bubbles in the silicone were reduced through oscillations or a vacuum. After the silicone had completely cured, it was cooled at 23 °C for around 6 h. Once cooled, the mould was removed, and the resulting silicone body was trimmed to the desired shape.

The upper side of the soft robot was sealed together with the upper cover plate with sealant to heat preservation. The contact end between the TEC and the soft robot was composed of fans, which could ensure a uniform temperature in the internal space of the soft robot as much as possible. At the same time, customized silica gel was used with a thermal conductivity of 0.7 W/m K. The cooling effect on the side close to the TEC was better, and the side away from the TEC was within 1 °C of the proximal end to meet the cooling requirement of the TPA.

**Figure 5:**
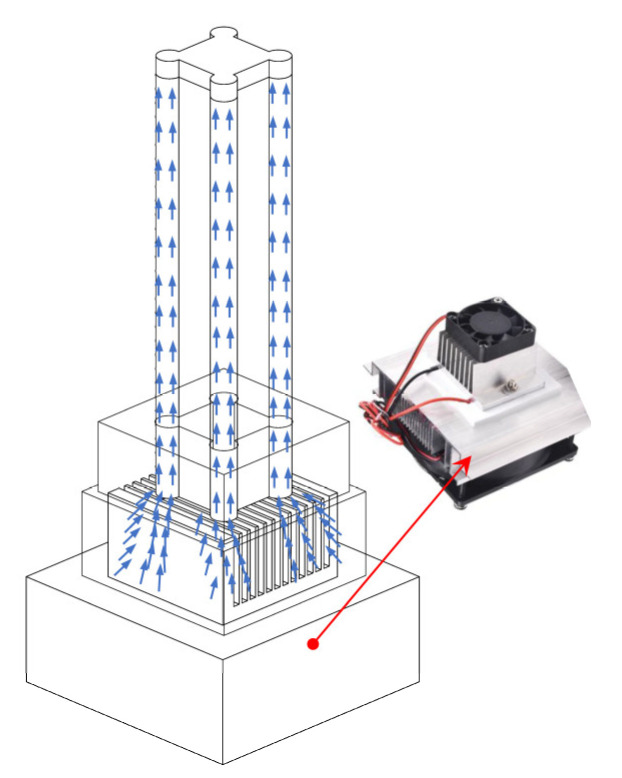
TEC cools the four TPAs in a soft robot.

The process of TEC cooling the TPAs is shown in Figure 5, where the TEC module and the soft robot were connected through a connecting module. The bottom of the soft robot was attached to the fan in the cooling sheet, and the cold air produced by the cooling sheet was evenly dispersed around the four TPAs by the fan. To optimize the cooling effect, the connection module was cemented to the soft robot and TEC with glue.

The motion mechanism of a single TPA was taken as an example. In the initial state, the motion angle of the robot was 0°, the driving temperature of the TPA was room temperature T_0_, and the length of the TPA was the initial length L_0_. When the pulse voltage was applied, the temperature of the TPA changed, and contractions occurred. The length of the TPA was reduced, driving the software and causing the robot to bend and deform. A soft robot driven by multiple TPAs can produce a variety of bending motions. This paper mainly discusses the motion performance of soft robots driven by two adjacent TPAs.

#### 2.2.2. Bending Deformation Modelling

**Figure 6:**
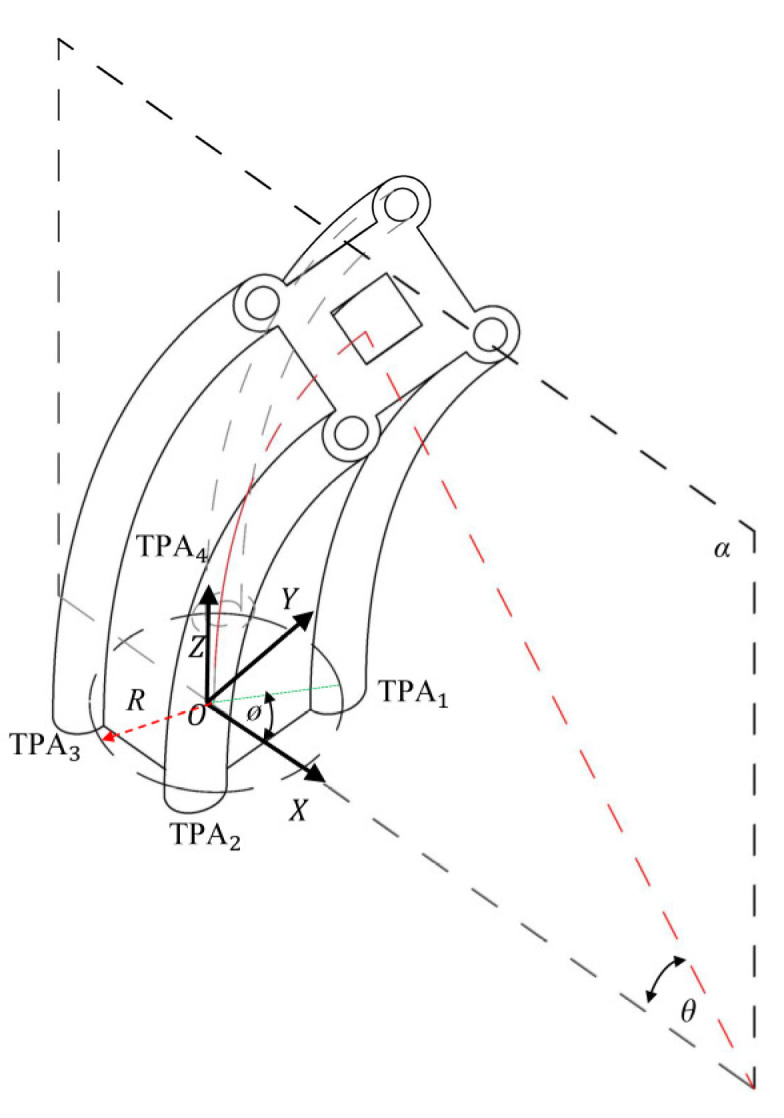
Analysis of the relationship between the robot bending angle and TPA length in space.

The bending deformation of the soft body was generated using four TPAs, and we considered the physical relationship between the bending deformation attitude and the deformation of the TPA. To describe this physical relationship, a simplified physical model of the structure was built. To assess the relationship between the TPA length and the bending angle of the soft robot, a coordinate system was established, as shown in Figure 6. The centre of the bottom surface was taken as the origin of the coordinate system; the bending direction driven by two TPAs was taken as the X-axis; the plane containing TPA1, TPA2, TPA3, and TPA4 was denoted as plane α; and the plane perpendicular to plane α was denoted as plane β.

**Figure 7:**
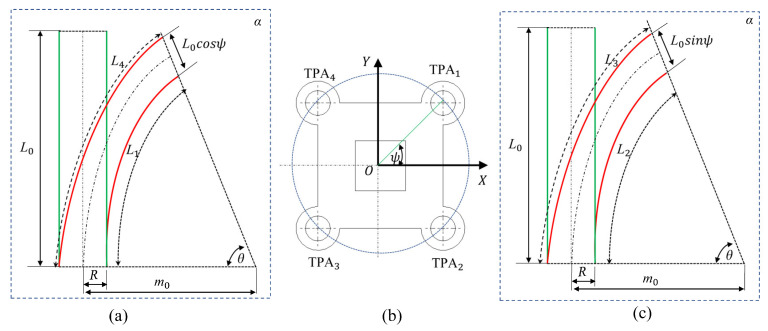
Analysis of the relationships between the bending angle and the TPA shrinkage length α plane: (**a**) projection view of TPA1 and TPA4 in the α plane; (**b**) elevation view of the bottom plane; (**c**) projection view of TPA2 and TPA3 in the α plane.

The initial length of the four TPAs was *L*_0_. When TPA1 and TPA3 received the pulse voltage signal, they shrank, while TPA2 and TPA4 were passively stretched. Therefore, the following relationships can be obtained according to Figure 7a,c:

(3) $$\left\{\begin{array}{l} L_{3}=(m_{0}-\frac{L_{0}}{2}\sin\psi )\theta \\ L_{4}=(m_{0}-\frac{L_{0}}{2}\cos\psi )\theta \end{array}\right.$$

where *L*_3_ and *L*_4_ indicate the lengths of TPA3 and TPA4 after Joule heating, respectively; *m*_0_ indicates the radius of the TPAs in the soft robot in space; *φ* is the bending angle with respect to the X-axis; and *θ* indicates the bending angle of the soft robot.

(4) $$\left\{\begin{array}{l} L_{1}=(m_{0}-\frac{L_{0}}{2}\sin\psi )\theta \\ L_{2}=(m_{0}-\frac{L_{0}}{2}\cos\psi )\theta \end{array}\right.$$

where *L*_1_ and *L*_2_ indicate the lengths of TPA1 and TPA2 after Joule heating, respectively.

#### 2.2.3. Control

**Figure 8:**
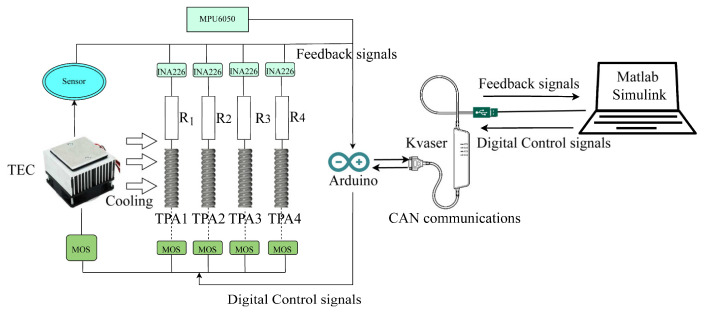
Control system.

For the angle measurement and temperature feedback functions of this system, an experimental system was established, as shown in Figure 8. This study used Simulink (MATLAB 2021b) for data transmission and processing. The real-time communication module Kvaser (USB to CAN interface, 1 Mbps output rate, 100 μs time accuracy) was used. An Arduino CAN transmits data from the temperature sensors and AD converters (Shen Zhen, China, Uxin Electronic Technology Company, Texas Instruments, INA226) to the Kvaser module. The pulse signal of the power supply is transmitted by the Arduino to a high-power MOS module, and the MOS module data is transmitted to the TEC and TPAs. The MPU6050 detects the angle change of the soft robot and realizes the closed-loop control of the robot by comparing this angle with the planned angle. 

**Figure 9:**
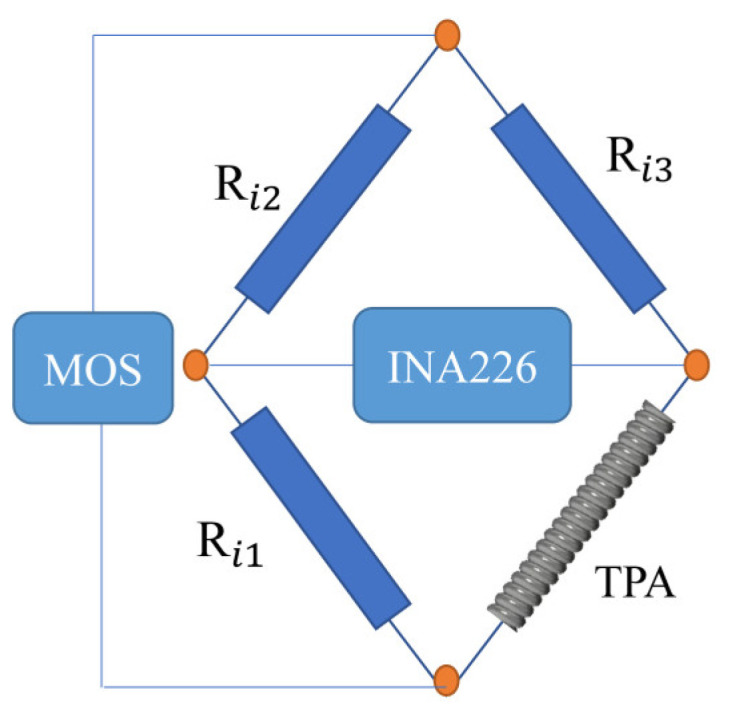
Wheatstone bridge measures the changing resistance.

Due to the low resolution of the AD converter in directly measuring the change in the TPA resistance, when the contraction bandwidth of the TPA increases, the detection error increases, affecting the measurement precision. In this paper, the unbalance principle of the Wheatstone bridge test thermistor was used to measure the resistance change in the TPA and improve the measurement precision, as shown in Figure 9.

**Figure 10:**
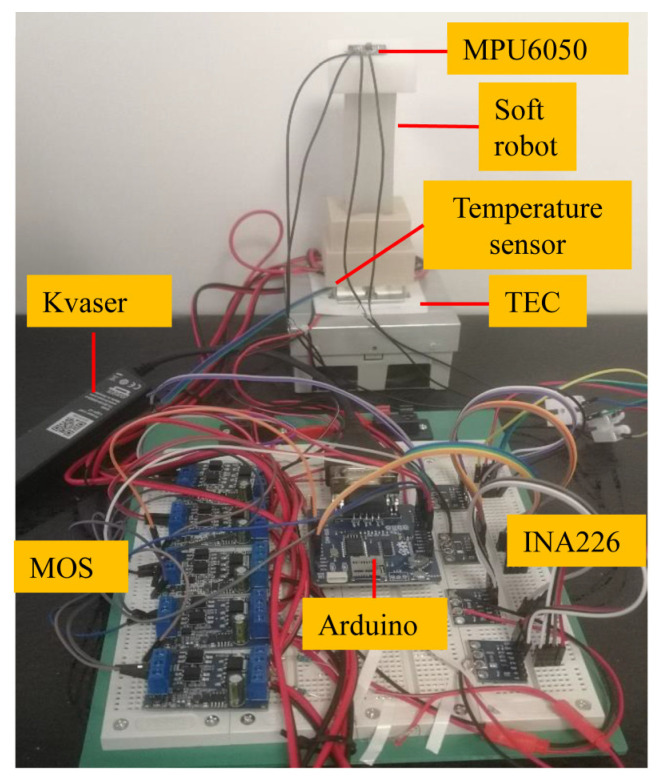
Prototype of the soft robot and its control system.

Figure 10 illustrates the prototype of the soft robot and its control system. The specific control process was designed as follows:

1. The temperature sensor detected the internal temperature of the soft robot. When the temperature was higher than 5 °C, the MOS tube connected to the Arduino controlled the TEC, which began cooling the system. When the temperature was lower than 5 °C, the input power to the MOS tube was stopped.
2. Based on the theoretical model, the input power corresponding to the target bending angle was determined, which was used as the first bending motion of the soft robot. The actual angle collected by the MPU6050 was compared with the planned value, the results were transmitted to the angle PID controller, and the target angle was realized by continuously increasing the input power.
3. When the actual angle was close to the target angle, the power transmitted to the contracted TPAs was gradually reduced. Then, the power to another pair of TPAs was increased to bend the robot in the opposite direction.
4. The TPA length changed as the power changed. Therefore, the TPA could control the soft robot to produce bending motion. In the sensing stage, due to natural cooling, the input power needed to be kept within 3 W, as the TPA could burn if the temperature was too high. When the TEC cooled the system, the input power needed to be kept within 20 W because the TPA was less likely to burn during cooling, and the motion frequency was increased.

## 3. Results

To verify the self-sensing ability of the TPA applied to soft robots and the bandwidth improvement effect of TEC cooling, we studied the sensing performance in the X-axis direction. Furthermore, the cooling effect of the TEC was verified using the motion of the soft robot at 0.5 Hz and 1 Hz.

### 3.1. Sensing

**Figure 11:**
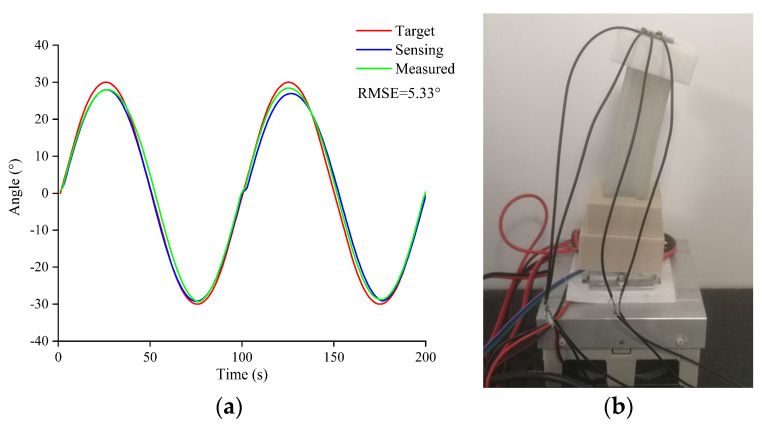
Sensing: (**a**) experiment under natural cooling at 0.01 Hz; (**b**) soft robot during its bending process.

Figure 11 shows the sinusoidal signal tracking response of the closed-loop control system of the joint bending angle. The system could track the robot over a period of 100 s in real time with only a small phase lag. The TPAs were heated for 50 s and naturally cooled for 50 s during motion. The *x*-axis ranged from −30° to 30°, and the maximum tracking error was 4.3°.

To evaluate the self-sensing performance of the TPA, sinusoidal signals were used to test the accuracy of the angle control system. The robot was bent in the positive and negative *x*-axis directions, as shown in Figure 11. The test results showed that the self-sensing effect of the TPA was good at a frequency of 0.01 Hz. The sensing angle curve approximately coincided with the angle measured using the MPU6050. The root-mean-square error was less than 5.33% of the measurement amplitude.

### 3.2. TEC Cooling

As the frequency increased, the power input to the TPA increased. When the power was too high, the TPA broke due to the high temperature, increasing the difficulty of closed-loop control of the soft robot at 0.5 Hz or 1 Hz. The TPAs were heated for 1 s and TEC cooled for 1 s during motion under 0.5 Hz. The TPAs were heated for 0.5 s and the TEC cooled for 0.5 s during motion under 1 Hz. At present, the soft robot can only move at a frequency of 0.5 Hz or 1 Hz and cannot carry out good closed-loop control.

**Figure 12:**
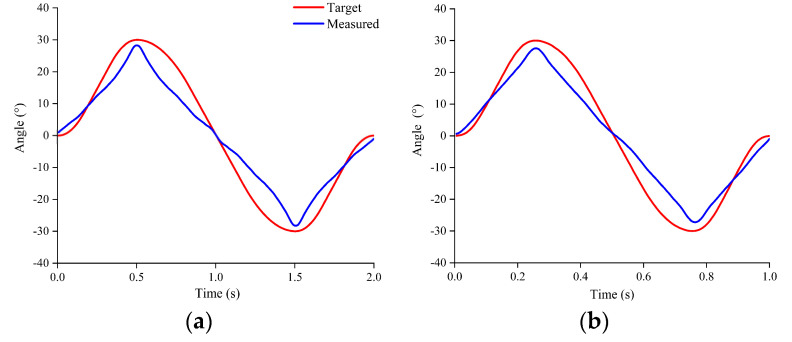
High-frequency motion under TEC cooling: (**a**) 0.5 Hz; (**b**) 1 Hz.

To study the effect of the TEC on the improvement in the motion frequency of the soft robot, the frequency was increased to 0.5 Hz and 1 Hz as shown in Figure 12. At these frequencies, the self-sensing ability of the TPA was poor, and thus, only motion experiments were conducted. As shown in Figure 11, the actual value could not move according to the planned curve, but the angular amplitude and movement frequency were essentially consistent with the planned values at 0.5 Hz. When the motion frequency was 1 Hz, the maximum angle difference reached 7.6°, and the frequency was essentially consistent with the planned value.

## 4. Discussion

TPAs show promising application value due to its shrinkage frequency, self-sensing ability, and large strain capability. The frequency of a TPA is directly affected by the cooling method. Although water cooling can greatly improve the frequency, the sealing and external water source requirements are inconvenient. Air cooling is the most widely used cooling method at present. Most air cooling methods use micro fans for refrigeration, but the refrigeration effect is not good, and it is difficult to substantially improve the movement frequency with this cooling approach.

In this study, the cooling effect of a TEC was simulated using air conditioning, and load experiments were conducted with a 2-ply TPA. The results showed that the TPA could reach a frequency of 3.3 Hz under cold air cooling, and the motion effect was stable. Based on these findings, a TEC was integrated into the soft robot. Wu et al. [30] indicated that finger joints can operate with a 20 s period (0.05 Hz frequency) without applying active cooling to the TPA. Sen et al. [31] built a prototype to test the grasping ability of a soft gripper at 0.013 Hz with passive cooling driven using a TPA. The test results showed that the robot could achieve a motion frequency of 1 Hz. However, due to the increase in the input power, angle control became more difficult, and the robot could not move according to the planned trajectory.

Due to the inherent properties of silver-coated nylon fibres, the variation in the resistance value with TPA shrinkage was tested in this study. In addition, the relationship between the angle of the soft robot and the change in length of the TPA was established to determine the change in the robot angle using the TPA. It is important to note that we revealed the self-sensing ability of the TPA by studying the relative resistance, not the absolute resistance. In this study, the TPA sensing ability was tested to obtain the angle change in the soft robot at a low frequency (0.01 Hz).

The self-sensing ability of TPAs in robot applications and their transient responses need to be understood to accurately determine the actuator position. When the robot’s motion frequency reached 1 Hz, its sensing ability was affected due to the transient response and potential creep in the resistance measurement throughout multiple cycles. The hysteresis, nonlinearity, and creep properties of the TPA directly affect its use in practical applications.

## 5. Conclusions

In this study, a TEC cooling method was proposed to improve the motion frequency of soft robots. Closed-loop control of the internal temperature of the soft robot was achieved with a temperature sensor and MOS. (1) A closed-loop control system with the bending angle as feedback was developed to realize a bending angle of 20° at frequencies of 0.5 Hz and 1 Hz. (2) To verify the self-sensing ability of the TPA, the relationship between the TPA length and resistance was first fit with a nonlinear function; then, the relationship between the bending angle of the soft robot and TPA length was established. The results verified that the TPAs had excellent sensing ability at 0.01 Hz.

## Data Availability

The datasets generated during and/or analyzed during the current study are available from the corresponding author upon reasonable request.
